# The Effects of Post-Mating Administration of Anti-IL-10 and Anti-TGFß on Conception Rates in Mice

**DOI:** 10.22074/ijfs.2015.4210

**Published:** 2015-04-21

**Authors:** Ali Risvanli, Ahmet Godekmerdan

**Affiliations:** 1 Department of Obstetrics and Gynecology, Faculty of Veterinary Medicine, University of Firat, Elazig, Turkey; 2 Deparment of Microbiology, Faculty of Medicine, University of Yildirim Beyazit, Ankara, Turkey

**Keywords:** Pregnancy, Mouse, Cytokine

## Abstract

**Background:**

In fertility studies, it has been shown that transforming growth factor β (TGFβ) and interlukin 10 (IL-10) play very important roles in implantation, maternal immune tolerance, placentation and fetal development, and the release beginning of release for fetal and postnatal death. The present study aims to determine the effects of the postmating administration of neutralizing antibodies against IL-10 and TGFβ, which significantly impact pregnancy in females and the conception rates in mice via assessments of blood serum and uterine fluid concentrations of IL-2, IL-4, IL-6, IL-10, IL-17, interferon γ (IFNγ), Tumor necrosis factor α (TNFα), and TGFβ.

**Materials and Methods:**

In this experimental study, 21 BALB/c strain female mice were mated and randomly divided into three groups. The mice in the first group were selected as the control group. The second group of animals was injected with 0.5 mg of anti-IL-10 after mating, while those in the third group were intraperitoneally injected with 0.5 mg of anti-TGFβ. The animals in all groups were decapitated on the 13thday after mating and their blood samples were taken. The uteri were removed to determine pregnancy. The mice’s uterine irrigation fluids were also obtained. We used the multiplex immunoassay technique to determine the cytokine concentrations in uterine fluid and blood serum of the mice.

**Results:**

We observed no intergroup difference with respect to conception rates. A comparison of the cytokine concentrations in the uterine fluids of pregnant mice revealed higher TGFβ concentrations (p<0.01) in the second group injected with the anti-IL-10 antibody compared with the other groups. There was no difference detected in pregnant animals with regards to both uterine fluid and blood serum concentrations of the other cytokines.

**Conclusion:**

Post-mating administration of anti-IL-10 and anti-TGFβ antibodies in mice may not have any effect on conception rates.

## Introduction

The maternal immune system plays an important role in establishment of pregnancy. It is generally believed that cellular immune response is inhibited while humoral immune response becomes dominant during gestation. In this regard, Treg cells and their secreted cytokines such as interlukin 10 (IL- 10) and transforming growth factor β (TGFβ) may have important roles.

The significant role played by Treg lymphocytes during pregnancy has been first shown in mice in 2004. Treg cells can be detected in lymph nodes that drain the uterus as early as two days after mating. It is also argued that there is an increase in the number of these cells within the days following mating in mice (1). Many different mechanisms have been suggested for the immunosuppressive effect of Treg lymphocytes. IL-10 and TGFβ are known to increase during this immunosuppression. IL-10 and TGFβ play very important roles in conception rates and the pregnancy period (2).

For a pregnancy, IL-10 and its receptors must be found in the endometrium and decidual cells in early pregnancy under normal conditions. This cytokine leads to the proliferation of decidual cells and the secretion of Tumor necrosis factor α (TNFα) due to the autocrine effect, while also causing a maternal immune response due to the paracrine effect (3).

TGFβ is known to play a role in providing maternal immune tolerance during implantation and in *in vitro* regulation of various implantation-related molecules such as vascular endothelial growth factor (VEGF), matrix metallopeptidase 9 (MMP-9), insulin-like growth factor-binding protein 1 (IGFBP-1), and leukemia inhabitory factor (LIF) (4, 5). Early embryonic deaths or postpartum deaths have been reported in TGFβ knockout mice (6). Other studies demonstrate mRNA expression in TGFβ type 1 and type 2 receptors in rat endometria during the estrous cycle and in early pregnancy, claiming that functional TGFβ signals are linked to the beginning of implantation and trophoblast invasion (7, 8).

Mating triggers a temporary inflammatory response in the uterus, particularly for reasons related to seminal plasma, which might start with blastocyst hatching and continue to implantation. This response is believed to originate from the immunopermissive effect of the lymphocytes in the uterus. Specific factors in the seminal vesicles such as TGFβ trigger cytokines and chemokines such as granulocyte macrophage colony-stimulating factor (GM-CSF) in the uterine epithelium and leukocytes in mice (9, 10). In this context, mating is also argued to cause immunomodulation in the long run. Prior to implantation, the embryo needs a suitable cytokine environment for survival. In particular, IL-10 with immunosuppressive activity has a role in the emergence of a mating-induced inflammatory response (11). Robertson et al. (12) have reported that IL-6 and GM-CSF levels also increased after mating.

The Treg lymphocyte population increases in regional lymph nodes and in the uterus of females exposed to seminal plasma as a result of mating. This increase is believed to play a role in the development of fetal immune tolerance prior to implantation. Moreover, TGFâ-involved immune deviation and antigenic stimulation should exist for this reaction to take place. This process requires both sperm and seminal plasma in the same environment (9, 13). The present study aims to determine the effects of post-mating administration of antibodies developed against IL-10 and TGFâ with roles in conception and continuation of pregnancy upon conception rates in mice. The blood serum and uterine fluid concentrations of IL-2, IL- 4, IL-6, IL-10, IL-17, interferon γ (IFNγ), TNFα, and TGFâ cytokines are also analyzed.

## Materials and Methods

### Animals

Female BALB/c mice, 3-4 months old, that weighed 25-30 g were used in this experimental study. The animals were obtained from the Experimental Research Center at Firat University. During the study period, the animals were kept in cages of seven animals per cage and were exposed to a light regimen of 12 hours dark and 12 hours light. They were given free access to food and water. The maintenance and care of experimental animals complied with the National Institutes of Health Guidelines for the Humane Use of Laboratory Animals or those of our Institute. A report was obtained from the Ethics Board for Experimental Animals of Firat University to conduct the study (FUHADEK No: 28/24.03.2010).

### Treatment groups

One male mouse was placed in each cage and the animals’ mating was monitored. Only females with vaginal plugs or those with spermatozoa in vaginal smears were considered to be mated females. The floors of all cages were covered with black material to facilitate the detection of vaginal plug formation. Vaginal plug monitoring was carried out at 2 hour intervals. This method was the preferred technique to avoid missing the vaginal plugs as an indication that mating had occurred. Then, the animals were randomly placed into three groups.

The animals in group 1 (n=7) were intraperitoneally injected with 0.5 ml of saline solution immediately after the detection of vaginal plugs or spermatozoa in vaginal smears.

The animals in group 2 (n=7) were intraperitoneally injected with 0.5 mg of monoclonal mouse anti-IL-10 antibody (eBioscience) immediately after the detection of vaginal plugs or spermatozoa in vaginal smears (14).

The animals in group 3 (n=7) were intraperitoneally injected with 0.5 mg of monoclonal mouse anti-TGFβ antibody (GeneTex) immediately after the detection of vaginal plugs or spermatozoa in vaginal smears (14).

The animals in all groups were decapitated 13 days after the detection of vaginal plugs or spermatozoa in vaginal smears. Before decapitation, their intracardiac blood was taken. Blood samples were centrifuged at 3000 rpm for 5 minutes and the resultant sera were stored at -80˚C until assayed. The uteri of the decapitated animals were removed to identify whether they were pregnant.

### Collection of uterine fluid

After the animals were killed, their uterine fluid was obtained as described by Harris et al. (15) and Orsi et al. (11). Accordingly, the cornua of the removed uteri were ligatured and then irrigated with mineral oil to collect uterine fluids. We used 1 ml of mineral oil for irrigation purposes. Subsequently these samples were microcentrifuged at 9000 rpm for 5 minutes to remove cell debris. The resultant uterine fluids were kept at -80˚C until the measurements were carried out.

### Cytokine analyses

The IL-2, IL-4, IL-6, IL-10, IL-17, IFNγ, TNFα, and TGFβ concentrations in blood serum samples and uterine fluids obtained from the animals were determined by multiplex immunoassay (Procarta ® Cytokine Assay Service, Diax, Italy) based on xMAP® technology. The multiplex assay is a test capable of a large number of simultaneous measurements. It is used in cases of small volumes of sera. All cytokines tested with immunoassay multiplex analysis can be made with a single plate. Since TGFβ kits can only determine the active form of TGFβ, acid assays have been performed to separate the active form of TGFβ in the samples. A separate plate was used for TGFβ measurements (16).

### Statistical analysis

The chi-squared test was used to compare intergroup conception rates while the Kruskal-Wallis test was performed to compare intergroup cytokine concentrations. In cases where the test showed significance, the Mann-Whitney U test was employed to determine the significance level. All statistical analyses were performed using SPSS 11.5 software.

## Results

Table 1 summarizes the conception rates on day 13 after mating in the light of the data obtained. No intergroup difference was detected in terms of conception rates (p>0.05).

A comparison of the cytokine concentrations in the uterine fluids from conceived animals revealed a significantly higher TGFâ concentration in group 2 injected with the anti-IL-10 antibody (12.30 ± 2.67 pg/ml) compared to the other groups (p<0.01, Table 2). However, no significant difference was found between conceived animals with regard to the concentrations of other cytokines both in uterine fluid and blood serum (p>0.05, Tables 2, 3).

Statistical computations were not performed for intergroup comparisons because the number of non-conceived animals was low in all groups. No comparison could be made between the cytokine concentrations of conceived and non-conceived animals due to the same reason.

**Table 1:** Distribution of pregnancy rates according to groups


Group 1 *		Group 2 *		Group 3 *	
Pregnancy		Pregnancy		Pregnancy	

**Positive**	**Negative**	**Positive**	**Negative**	**Positive**	**Negative**
N (%)	N (%)	N (%)	N (%)	N (%)	N (%)
6 (85.71)	1 (14.29)	5 (71.43)	2 (28.57)	5 (71.43)	2 (28.57)


No significant differences between groups (p>0.05), *; N=7.

**Table 2:** Distribution of uterine fluid cytokine concentrations according to groups


Cytokine(pg/ml)	Group 1		Group 2		Group 3		P
	(n=7)		(n=7)		(n=7)		
	Pregnancy		Pregnancy		Pregnancy		
	+	-	+	-	+	-	
	(n=6)	(n=1)	(n=5)	(n=2)	(n=5)	(n=2)	

**IL-2**	4.33±0.49	4.0	3.30±0.49	4.5	2.80±0.20	3.0	-
**IL-4**	3.83±0.65	4.0	4.00±0.77	3.0	3.80±0.37	3.5	-
**IL-6**	5.50±1.12	5.0	5.00±0.89	5.5	4.80± 0.97	4.5	-
**IL-10**	3.33±0.21	3.0	4.30±0.44	4.25	5.20± 0.97	3.5	-
**IL-17**	7.33±0.71	6.0	7.00±0.32	7.00	6.40± 1.02	6.0	-
**IFNγ**	38.83±5.87	53.0	48.17±3.32	48.75	49.00±4.37	60.5	-
**TNFα**	4.67±0.49	6.0	4.60±0.51	4.5	3.90± 0.40	4.75	-
**TGFβ**	4.66±0.33^b^	6.0	12.30±2.67^a^	17.5	5.50± 0.61^b^	4.75	*


-; No significant differences between groups (p>0.05). a, b; The difference between different letter-carrying averages is significant, *; P<0.01, IL; interlukin, IFNγ; interferon γ, TNFα; Tumor necrosis factor α and TGFβ; transforming growth factor β.

**Table 3:** Distribution of serum cytokine concentrations according to groups


Cytokine(pg/ml)	Group 1		Group 2		Group 3		P
	(n=7)		(n=7)		(n=7)		
	Pregnancy		Pregnancy		Pregnancy		
	+	-	+	-	+	-	
	(n=6)	(n=1)	(n=5)	(n=2)	(n=5)	(n=2)	

**IL-2**	6.17±1.04	14.0	4.4±0.43	6.5	5.41± 2.22	3.75	-
**IL-4**	10.92±5.29	31.0	7.00±1.17	9.0	9.34± 13.17	6.5	-
**IL-6**	63.11±7.88	87.0	38.40± 25.05	12.0	74.80±17.44	127.75	-
**IL-10**	5.0±0.73	4.0	4.8±0.2	4.0	5.2±0.58	3.5	-
**IL-17**	287.73±11.12	195.0	170.90±56.58	168.5	218.80±27.60	396.0	-
**IFNγ**	26.33±5.12	18.0	24.20± 4.0	19.0	5.33± 5.21	14.75	-
**TNFα**	31.25±8.52	14.5	39.33± 12.33	32.5	19.50±4.84	14.5	-
**TGFβ**	2403.08±410.56	2830.5	3798.50±962.25	4177.5	2287.60±710.70	4147.25	-


-; No significant differences between groups (p>0.05), IL; interlukin, IFNγ; interferon γ, TNFα; Tumor necrosis factor α and TGFβ; transforming growth factor β.

## Discussion

Studies that examine the role of cytokines in reproductive processes have argued that IL-10 has a role in fertility, implantation, maternal immune tolerance, placentation, and fetal development (3), while the same roles are also played by TGFβ and fetal or postpartum deaths are claimed to occur due to the defects in its release (6).

Immunosuppressive IL-10 and TGFβ secreted by Treg cells during pregnancy inhibit the effector functions of activated leukocytes (17, 18). Slager et al. (19) have reported a considerable decrease in implantation rates after the neutralizing antibodies specific for TGFβ-2 were injected into the cavity of mouse blastocysts 3.5 days after mating. After one day in culture, embryos were transferred to pseudopregnant females. In order to evaluate the physiologic roles of IL-10, BALB/c mice were continuously treated with neutralizing anti-IL-10 antibodies from birth until the eighth week as follows: three times per week mice received 0.2 mg/injection for week one, 0.5 mg/injection for week two, and 1.0 mg/injection for weeks three through eight. As a result, their endogenous IFNγ and TNFα levels increased (20). In a study aimed at eliminating the protective effects of Treg cells in pregnant mice (14), anti-IL-10 antibodies were used as a 1 mg single intraperitoneal dose. Very high abortion rates were observed in the animals injected with these antibodies. In the same study, the researchers blocked TGFβ in pregnant animals by using neutralizing antibodies administered as a single 1 mg intraperitoneal dose. They observed high abortion rates in these animals as well, although not as high as in the groups treated with anti-IL-10 antibodies. This result has demonstrated the importance of TGFβ and particularly IL-10 secreted by Treg cells during pregnancy in protecting the allogeneic fetus. The results of the present study were not consistent with the results of these studies. These differences might arise from issues such as the method antibody administration, breed discrimination, and dosage.

There are certain publications asserting views that are contrary to those presented above. For instance, Rijhsinghani et al. (21) have treated pregnant mice with anti-IL-10 monoclonal antibodies to neutralize IL-10. As a result they found that this treatment did not affect pregnancy duration and there were no adverse effects on fetal development. However, certain problems in the newborn at later stages were observed. It has been reported that in early pregnancy and during implantation, inflammatory responses develop in endometrial tissues of IL-10 null mutant mice following mating despite inadequate IL-10. The implantation rates in these animals and survival rates of their newborns are higher when compared with normal mice. Thus, these studies have claimed that IL-10 is not required in mice for maternal immune tolerance and for a healthy pregnancy. In the present study, we observed that the post-mating anti-TGFβ and anti-IL-10 treatments in mice did not have any effect on conception rates. In this respect, the data obtained were consistent with the results reported in the above mentioned studies.

Conflicting results have been obtained in various studies with regard to the impacts of anti-IL-10 and anti-TGFβ treatments on the formation and continuation of pregnancy. Nevertheless, fetal formation and survival requires the development of maternal immunotolerance during pregnancy and this requires an immunosuppressive environment, also including IL-10 and TGFβ. At this point, neutralizing antibodies that develop against IL-10 and TGFβ may adversely affect the formation and continuation of pregnancy by suppressing the functions of the cytokines in question. Studies have been conducted by researchers opposing this hypothesis such as Rijhsinghani et al. (21) and White et al. (22) that failed to mention whether the antibodies used sufficiently neutralized IL-10 and TGFβ. They also varied in their methods used as well as the number and types of their subjects. Researchers who proposed positive opinions about the subject used different concentrations of anti-IL-10 and anti- TGFβ during pregnancy. No study has been found on the post-mating use of these antibodies.

In the present study, despite the lack of any statistical difference, the blood serum TNFα and TGFβ concentrations were found to be higher in the group treated with anti-IL-10 antibodies compared with the other groups, whereas IL-2, IL-4, IL-6, IL-10, and IL-17 were lower in this group. Even though there was no statistical difference, this result could be interpreted to demonstrate that treatment of these antibodies in mice partially influenced blood serum cytokine concentrations in pregnant animals. However, since a limited number of animals were used in the study for ethical reasons we could not compare cytokine concentrations in conceived and non-conceived animals. Hence, we did not have any findings on this topic. Similar interpretations also apply to the group treated with anti-TGFβ antibodies and could be extended to comparisons of both uterine fluid and blood serum cytokine concentrations between conceived and nonconceived animals.

One of the main objectives of reproductive biology is a healthy early pregnancy and, in particular, implantation period. The present study has aimed at demonstrating the importance of IL-10 and TGFβ in the formation of pregnancy and attempted to prevent pregnancy formation in mice by the post-mating use of neutralizing antibodies developed against these cytokines. In this way, this study also aimed to obtain data to develop a new immunocontraceptive method to control reproduction. However, in our study, we observed that either post-mating anti-TGFβ and or anti- IL-10 treatments in mice did not influence conception rates. Therefore, we concluded that it would be useful to support these results with further studies that use a greater number of subjects and/or other species.

## Conclusion

In the present study, we observed that cytokine concentrations obtained from the uterine fluid were significantly lower than the blood serum cytokine concentrations in all groups. However, our results were consistent with the findings of Orsi et al. (11) and these conclusions should be considered by studies that aim to determine cytokine concentrations and their effects in organs with lumen, such as the uterus. The lower cytokine concentrations in uterine fluid compared with blood serum could be attributed to the fact that cytokine concentrations might have been diluted during the irrigation process.
